# The Advisory Committee on Immunization Practices’ Interim Recommendation for Use of Pfizer-BioNTech COVID-19 Vaccine — United States, December 2020

**DOI:** 10.15585/mmwr.mm6950e2

**Published:** 2020-12-18

**Authors:** Sara E. Oliver, Julia W. Gargano, Mona Marin, Megan Wallace, Kathryn G. Curran, Mary Chamberland, Nancy McClung, Doug Campos-Outcalt, Rebecca L. Morgan, Sarah Mbaeyi, José R. Romero, H. Keipp Talbot, Grace M. Lee, Beth P. Bell, Kathleen Dooling

**Affiliations:** ^1^CDC COVID-19 Response Team; ^2^Epidemic Intelligence Service, CDC; ^3^General Dynamics Information Technology, Falls Church, Virginia; ^4^University of Arizona College of Medicine, Phoenix, Arizona; ^5^Department of Health Research Methods, Evidence, and Impact, Hamilton, Ontario; ^6^Arkansas Department of Health; ^7^Vanderbilt University School of Medicine, Nashville, Tennessee; ^8^Stanford University School of Medicine, Stanford, California; ^9^University of Washington, Seattle, Washington.

On December 11, 2020, the Food and Drug Administration (FDA) issued an Emergency Use Authorization (EUA) for the Pfizer-BioNTech COVID-19 (BNT162b2) vaccine (Pfizer, Inc; Philadelphia, Pennsylvania), a lipid nanoparticle-formulated, nucleoside-modified mRNA vaccine encoding the prefusion spike glycoprotein of SARS-CoV-2, the virus that causes coronavirus disease 2019 (COVID-19) (*1*). Vaccination with the Pfizer-BioNTech COVID-19 vaccine consists of 2 doses (30 *μ*g, 0.3 mL each) administered intramuscularly, 3 weeks apart. On December 12, 2020, the Advisory Committee on Immunization Practices (ACIP) issued an interim recommendation* for use of the Pfizer-BioNTech COVID-19 vaccine in persons aged ≥16 years for the prevention of COVID-19. To guide its deliberations regarding the vaccine, ACIP employed the Evidence to Recommendation (EtR) Framework,^†^ using the Grading of Recommendations, Assessment, Development and Evaluation (GRADE) approach.^§^ The recommendation for the Pfizer-BioNTech COVID-19 vaccine should be implemented in conjunction with ACIP’s interim recommendation for allocating initial supplies of COVID-19 vaccines (*2*). The ACIP recommendation for the use of the Pfizer-BioNTech COVID-19 vaccine under EUA is interim and will be updated as additional information becomes available.

Since June 2020, ACIP has convened nine public meetings to review data on the epidemiology of COVID-19 and the potential use of COVID-19 vaccines, including the Pfizer-BioNTech COVID-19 vaccine (*3*). Within the EtR Framework, ACIP considered the importance of the public health problem of COVID-19, as well as issues of resource use, benefits and harms, patients’ values and preferences, acceptability, feasibility, and equity for the Pfizer-BioNTech COVID-19 vaccine. To inform the EtR Framework, the COVID-19 Vaccines Work Group, comprising experts in infectious disease, vaccinology, vaccine safety, public health, and ethics, held 27 meetings to review COVID-19 surveillance data, evidence for vaccine efficacy and safety, and implementation considerations for COVID-19 vaccines, including the Pfizer-BioNTech COVID-19 vaccine. After a systematic review of the literature, the Work Group used the GRADE approach to assess the certainty of evidence for outcomes related to the vaccine, rated on a scale of 1 (high certainty) to 4 (very low certainty) (*4*). Work Group conclusions regarding the evidence for the Pfizer-BioNTech COVID-19 vaccine were presented to ACIP at public meetings.

The body of evidence for the Pfizer-BioNTech COVID-19 vaccine was primarily informed by one large, randomized, double-blind, placebo-controlled Phase II/III clinical trial that enrolled >43,000 participants (median age = 52 years, range = 16–91 years) (*5*,*6*). Interim findings from this clinical trial, using data from participants with a median of 2 months of follow-up, indicate that the Pfizer-BioNTech COVID-19 vaccine was 95.0% effective (95% confidence interval = 90.3%–97.6%) in preventing symptomatic laboratory-confirmed COVID-19 in persons without evidence of previous SARS-CoV-2 infection. Consistent high efficacy (≥92%) was observed across age, sex, race, and ethnicity categories and among persons with underlying medical conditions as well as among participants with evidence of previous SARS-CoV-2 infection. Although numbers of observed hospitalizations and deaths were low, the available data were consistent with reduced risk for these severe outcomes among vaccinated persons compared with that among placebo recipients. Among vaccine recipients, reactogenicity symptoms, defined as solicited local injection site or systemic reactions during the 7 days after vaccination, were frequent and mostly mild to moderate. Systemic adverse reactions were more commonly reported after the second dose than after the first dose and were generally more frequent and severe in persons aged 18–55 years than in those aged >55 years. Systemic adverse reactions had a median onset of 1–2 days after vaccine receipt and resolved in a median of 1 day. Severe local and systemic adverse reactions (grade ≥3, defined as interfering with daily activity) occurred more commonly in vaccine recipients than in placebo recipients. Among vaccine recipients, 8.8% reported any grade ≥3 reaction; the most common symptoms were fatigue (4.2%), headache (2.4%), muscle pain (1.8%), chills (1.7%), and injection site pain (1.4%). Generally, grade ≥3 reactions were more commonly reported after the second dose than after the first dose and were less prevalent in older than in younger participants. Serious adverse events^¶^ were observed in a similar proportion of vaccine (0.6%) and placebo (0.5%) recipients and encompassed medical events occurring at a frequency similar to that within the general population (*6*). No specific safety concerns were identified in subgroup analyses by age, race, ethnicity, underlying medical conditions, or previous SARS-CoV-2 infection. A detailed summary of safety data, including information on reactogenicity, is available at https://www.cdc.gov/vaccines/covid-19/info-by-manufacturer/pfizer/reactogenicity.html.

From the GRADE evidence assessment, the level of certainty for the benefits of the Pfizer-BioNTech COVID-19 vaccine was type 1 (high certainty) for the prevention of symptomatic COVID-19. Evidence was type 3 (low certainty) for the estimate of prevention of COVID-19–associated hospitalization and type 4 (very low certainty) for the estimate of prevention of death. Data on hospitalizations and deaths are limited at this time, but a vaccine that effectively prevents symptomatic infection is expected to also prevent hospitalizations and deaths. Regarding potential harms after vaccination, evidence was type 2 (moderate certainty) for serious adverse events and type 1 (high certainty) for reactogenicity. No data were available to assess the efficacy for prevention of asymptomatic SARS-CoV-2 infection. Data reviewed within the EtR Framework supported the use of the Pfizer-BioNTech COVID-19 vaccine. ACIP determined that COVID-19 is a major public health problem and that use of the Pfizer-BioNTech COVID-19 vaccine is a reasonable and efficient allocation of resources. Whereas there might be uncertainty in how all populations value the vaccine, it was determined that for most populations, the desirable effects outweigh the undesirable effects. The vaccine is probably acceptable to implementation stakeholders and feasible to implement in spite of difficult ultracold-chain storage and requirements for handling and administration. These requirements could limit the availability of the Pfizer-BioNTech COVID-19 vaccine to some populations thereby negatively impacting health equity. Therefore, efforts should be made to overcome these challenges and advance health equity. The GRADE evidence profile and EtR supporting evidence are available at https://www.cdc.gov/vaccines/acip/recs/grade/covid-19-pfizer-biontech-vaccine.html and https://www.cdc.gov/vaccines/acip/recs/grade/covid-19-pfizer-biontech-etr.html.

Before vaccination, the EUA Fact Sheet should be provided to recipients and caregivers. Providers should counsel Pfizer-BioNTech COVID-19 vaccine recipients about expected systemic and local reactogenicity. Additional clinical considerations, including details of administration and use in special populations (e.g., persons who are pregnant or immunocompromised or who have severe allergies) are available at https://www.cdc.gov/vaccines/covid-19/info-by-manufacturer/pfizer/clinical-considerations.html Additional studies of safety and effectiveness are planned after authorization and will be important to inform future ACIP recommendations as well as increase public confidence in the COVID-19 vaccination program. The interim recommendation and clinical considerations are based on use of the Pfizer-BioNTech COVID-19 vaccine under an EUA and might change as more evidence becomes available. ACIP will continue to review additional data as they become available; updates to recommendations or clinical considerations will be posted on the ACIP website (https://www.cdc.gov/vaccines/hcp/acip-recs/vacc-specific/covid-19.html).

## Reporting of Vaccine Adverse Events

Adverse events that occur in a recipient after receipt of COVID-19 vaccine should be reported to the Vaccine Adverse Events Reporting System (VAERS). FDA requires that vaccination providers report vaccination administration errors, serious adverse events, cases of multisystem inflammatory syndrome, and cases of COVID-19 that result in hospitalization or death after administration of COVID-19 vaccine under EUA. Reporting is encouraged for any clinically significant adverse event, whether or not it is clear that a vaccine caused the adverse event. Information on how to submit a report to VAERS is available at https://vaers.hhs.gov/index.html or 1-800-822-7967. In addition, CDC has developed a new, voluntary smartphone-based tool, v-safe, that uses text messaging and web surveys to provide near real-time health check-ins after patients receive COVID-19 vaccination. The CDC/v-safe call center follows up on reports to v-safe that indicate a medically significant health impact to collect additional information for completion of a VAERS report. Information on v-safe is available at https://www.cdc.gov/vsafe.

**Summary**

**What is already known about this topic?**

On December 11, 2020, the Food and Drug Administration issued an Emergency Use Authorization for the Pfizer-BioNTech COVID-19 vaccine.

**What is added by this report?**

On December 12, 2020, after an explicit, evidence-based review of all available data, the Advisory Committee on Immunization Practices (ACIP) issued an interim recommendation for use of the Pfizer-BioNTech COVID-19 vaccine in persons aged ≥16 years for the prevention of COVID-19.

**What are the implications for public health practice?**

The recommendation for the Pfizer-BioNTech COVID-19 vaccine should be implemented in conjunction with ACIP’s interim recommendation for allocating initial supplies of COVID-19 vaccines.

## Footnotes

* On December 12, 2020, ACIP voted 11–0 (three recusals) in favor of the interim recommendation for use of Pfizer-BioNTech COVID-19 vaccine. Three ACIP members recused themselves because of participation in clinical trials and/or other studies involving companies producing COVID-19 vaccines.

† https://www.cdc.gov/vaccines/acip/recs/grade/downloads/ACIP-evidence-rec-frame-508.pdf.

§ https://www.cdc.gov/vaccines/acip/recs/grade/about-grade.html.

¶ Serious adverse events are defined as any untoward medical occurrence that results in death, is life-threatening, requires inpatient hospitalization or prolongation of existing hospitalization, or results in persistent disability/incapacity.
