# *Erratum:* Vol. 69, No. 50

**DOI:** 10.15585/mmwr.mm7004a5

**Published:** 2021-01-29

In the report “The Advisory Committee on Immunization Practices’ Interim Recommendation for Use of Pfizer-BioNTech COVID-19 Vaccine — United States, December 2020,” on page 1922, in the third paragraph, the third and fourth sentences should have read **Consistent high efficacy (≥92%) was observed across age, sex, race, and ethnicity categories and among persons with underlying medical conditions. Efficacy was similarly high in a secondary analysis including participants both with or without evidence of previous SARS-CoV-2 infection.** Although numbers of observed hospitalizations and deaths were low, the available data were consistent with reduced risk for these severe outcomes among vaccinated persons compared with that among placebo recipients.

